# Varying Auxin Levels Induce Distinct Pluripotent States in Callus Cells

**DOI:** 10.3389/fpls.2018.01653

**Published:** 2018-11-13

**Authors:** Jinwoo Shin, Pil Joon Seo

**Affiliations:** ^1^Department of Biological Sciences, Sungkyunkwan University, Suwon, South Korea; ^2^Department of Chemistry, Seoul National University, Seoul, South Korea; ^3^Plant Genomics and Breeding Institute, Seoul National University, Seoul, South Korea

**Keywords:** auxin, callus, cellular reprogramming, cytokinin, plant regeneration, pluripotency

Plants have evolved a remarkable ability for reprogramming cell identity to facilitate tissue repair and developmental plasticity. During cell fate transition, an unorganized pluripotent cell mass, called a callus, is formed. Two major types of calli are observed depending on the inductive external stimuli: mechanical wounding alone or wounding followed by incubation on high auxin-containing callus-inducing medium (CIM) (Ikeuchi et al., 2013). Wound-induced calli and CIM-induced calli are similar in having pluripotency that ensures competence for subsequent tissue regeneration. However, the identity of pluripotency in wound-induced calli and CIM-induced calli is not analogous.

Wound-induced calli derive from various cell types and regenerate new tissues. Mechanical wounding without exogenous auxin treatment primarily activates cytokinin signaling and induces minimal callus cells at excised regions (Iwase et al., 2011). An AP2/ERF transcription factor, WOUND-INDUCED DEDIFFERENTIATION 1 (WIND1), and its close homologs, WIND2, WIND3, and WIND4, are central regulators of wound-induced callus formation in *Arabidopsis* (Iwase et al., 2011). Dominant-negative repression of *WIND*s, *WIND1-SRDX*, results in reduced callus formation in hypocotyl explants (Iwase et al., 2011), whereas overexpression of individual *WIND* genes is sufficient to induce calli from somatic cells (Iwase et al., 2011). The WIND proteins act through type-B ARABIDOPSIS RESPONSE REGULATORs (ARRs), which are key components of cytokinin signaling, and consistently, WIND activation of callus formation is strongly repressed in *arr1 arr12* double mutants (Iwase et al., 2011).

Upon endogenous activation of cytokinin signaling, concomitant with basal auxin biosynthetic activity, cell fate can be reversibly and freely switched. A low barrier of cell fate changes may lead to the coexistence of bipotent stem cells in callus tissues, which can give rise to either a root or shoot fate. This intermediate phase allows flexible tissue regeneration depending on the adjacent cells. Indeed, wound-induced calli are optimized for tissue repair. Neighboring cells, which are in direct contact with damaged regions, rapidly activate the ETHYLENE RESPONSE FACTOR 115 (ERF115)-PHYTOCHROME A SIGNAL TRANSDUCTION 1 (PAT1) complex to replenish collapsed cells through active reentry into the cell cycle. The ERF115-PAT1 complex possibly promotes *WIND1* expression by directly binding to the gene promoter (Heyman et al., 2016). WIND1 further regulates cytokinin-dependent cell division and tissue regeneration (Iwase et al., 2017), facilitating rapid damage healing (Figure 1). Despite a high efficiency of tissue repair, *WIND1*-overexpressing calli show reduced ability of *de novo* organogenesis without incubation on CIM (Iwase et al., 2017), reinforcing the optimized role of wound-induced calli in tissue repair.

**Figure 1:**
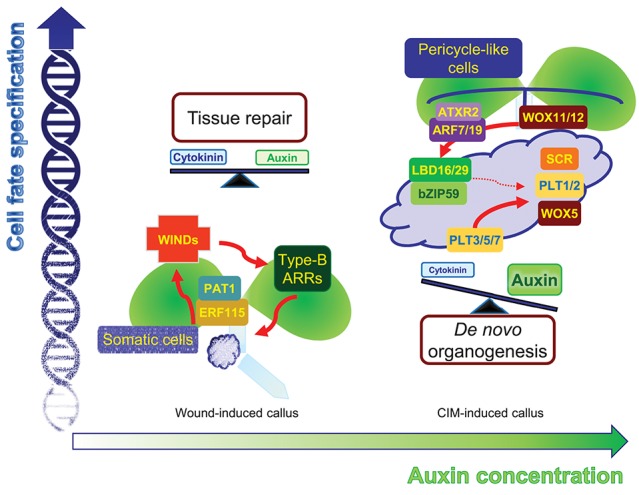
Comparison of two different types of callus. Mechanical wounding alone induces WIND1-type B ARR module-dependent cytokinin signaling as well as leaf-derived auxin accumulation. Moderate activation of both cytokinin and auxin signaling facilitates the formation of intermediate pluripotent cells in calli, which ensure rapid tissue repairs depending on the neighboring cell environment. The application of high auxin, in addition to wounding, induces another type of pluripotency in the callus, which is similar to root primordium. The regions expressing molecular components in the callus, which define root stem cell niche, may possess a genuine pluripotency that allows planned *de novo* organogenesis with efficient cell division activity.

High concentrations of auxin, in addition to mechanical wounding, result in another level of cell fate changes. Unlike wound-induced calli that do not display particular tissue identity, CIM-induced calli are similar to root primordium irrespective of the origin of the explants (Atta et al., 2009; Sugimoto et al., 2010). Although the calli are not genuine root primordia themselves, the calli have a gene expression profile similar to that found in root primordia and a genetic circuit of callus formation and root primordium establishment is significantly overlapped. On CIM, a pluripotent callus is usually induced from pericycle cells (or pericycle-like cells of aerial tissues) adjacent to the xylem poles though asymmetric or formative divisions (Valvekens et al., 1988; Atta et al., 2009), similar to lateral root emergence. Consistently, auxin signaling components mediating lateral root initiation are also involved in CIM-induced callus formation. The ARABIDOPSIS TRITHORAX-RELATED 2 (ATXR2)-AUXIN RESPONSE FACTOR (ARF)-LATERAL ORGAN BOUNDARIES DOMAIN (LBD) axis is a crucial signaling scheme underlying lateral root formation as well as callus formation (Okushima et al., 2007; Fan et al., 2012; Lee et al., 2017). ATXR2 interacts with ARF7 and ARF19, and the ATXR2-ARF complex specifically binds to the *LBD16* and *LBD29* promoters and activates expression through deposition of the active H3K36me3 mark, stimulating proliferation of pericycle competent cells and conferring root primordium characteristics in the callus (Okushima et al., 2007; Lee et al., 2017) (Figure 1). Accordingly, CIM-induced callus formation is impaired in leaf explants of *atxr2* and *arf7arf19* mutants and *LBD-SRDX* transgenic plants (Fan et al., 2012; Lee et al., 2017).

Following acquisition of root primordium characteristics in calli, many root stem cell regulators including WUSCHEL-RELATED HOMEOBOX 5 (WOX5), SCARECROW (SCR), PLETHORA 1 (PLT1), and PLT2 are expressed in callus cells (Atta et al., 2009; Kareem et al., 2015), although they have relatively broad spatial expression in the callus, rather than a confined expression into specific cell types. The histone acetyltransferase HISTONE ACETYLTRANSFERASE OF THE GNAT FAMILY 1 (HAG1)/ GENERAL CONTROL NONDEREPRESSIBLE 5 (GCN5) transcriptionally activates root-meristem genes in calli, including *WOX5, WOX14, SCR, PLT1*, and *PLT2*, establishing competence for subsequent *de novo* organogenesis (Kim et al., 2018). In addition, miRNA-directed ARF regulation is also involved in this process. The miR160 represses pluripotency acquisition during callus formation through mRNA cleavage of *ARF10*, which controls differentiation of distal root stem cells by defining spatial expression patterns of root stem cell regulators, such as *WOX5* and *PLT*s, as well as a cytokinin signaling gene *ARABIDOPSIS RESPONSE REGULATOR 15* (*ARR15*) (Ding and Friml, 2010; Liu et al., 2016). Moreover, the LBD-bZIP59 complex further directly regulates the *FAD-binding Berberine* (*FAD-BD*) gene to facilitate pluripotency establishment in calli (Xu et al., 2018). Cell clustering that has similarity to the root stem cell niche may be the identity of pluripotency in CIM-induced calli, ensuring competence for *de novo* organogenesis (Liu et al., 2018a).

Consistent with the fact that callus tissues have similarity to root primordium, *de novo* root organogenesis can spontaneously occur from callus cells especially at a lower concentration of exogenous auxin (Yu et al., 2017). In support, significant overlap of molecular components between *de novo* root organogenesis and callus formation have been demonstrated (Liu et al., 2014, 2018b; Lee et al., 2018). Notably, *de novo* shoot organogenesis can also be derived from CIM-induced calli. Since molecular components and networks in the stem cell niche of the shoot and root are well conserved (Sarkar et al., 2007; Rosspopoff et al., 2017), callus cells expressing root meristem regulators may be efficiently converted into shoot meristem upon incubation on shoot-inducing medium (SIM). Consistently, *lbd16* single and *plt3/5/7* triple mutants, which impair root meristem specification, show an inability for not only root organogenesis but also shoot regeneration from CIM-induced calli (Kareem et al., 2015). In addition, this type of pluripotency facilitates stepwise *de novo* organogenesis during *in vitro* plant regeneration. Upon transfer of calli preincubated on CIM to SIM, transcript levels of root stem cell regulators promptly decline. However, it is noteworthy that shoot stem cell regulators, WUSCHEL (WUS) and SHOOT MERISTEMLESS (STM), are slowly induced, rather than being rapidly activated, in a confined domain in response to high cytokinin (Gordon et al., 2007). A clear lag phase is established between the peak expression of root and shoot stem cell regulators, and this phase prevents reversible cell fate switching, directing planned tissue regeneration.

A remaining question would be what characteristics of the root primordium-like tissues are advantageous to calli for pluripotency. Accumulating evidence has suggested that the root primordium has an efficient cell proliferation system. Callus cells originate from the pericycle cells, which have low endoreduplication activity and thereby active cell division (Blakely and Evans, 1979). Endoreduplicated cells with high DNA contents have reduced regenerative potential and low genome integrity (Torrey, 1967). In support, CIM-induced calli, which begin with pericycle cells, ensure not only division activity but also genome integrity, while wound-induced calli allow high frequency regeneration of polyploid shoots (Torrey, 1967). Moreover, photosynthetic activity most likely interferes with pluripotent callus formation. Extensive light reactions require expensive reactive oxygen species (ROS) scavenging systems to deal with excessive endogenous free radicals. Given the trade-off between ROS metabolism and cell proliferation, plants have likely evolved to undergo transition into the root meristem during callus formation to enable active cell division.

Overall, unlike wounding alone, high auxin plus wounding provides limited flexibility of cell fate transition and thereby ensures stepwise transition of cell identity. This facilitates planned *de novo* organogenesis, which is required for the *in vitro* tissue culture process. This type of pluripotency, which resembles the root primordium, possesses a genuine competence for tissue regeneration with a cost-effective cell division process.

## Author contributions

All authors listed have made a substantial, direct and intellectual contribution to the work, and approved it for publication.

### Conflict of interest statement

The authors declare that the research was conducted in the absence of any commercial or financial relationships that could be construed as a potential conflict of interest.
